# A179L, a viral Bcl-2 homologue, targets the core Bcl-2 apoptotic machinery and its upstream BH3 activators with selective binding restrictions for Bid and Noxa

**DOI:** 10.1016/j.virol.2008.01.050

**Published:** 2008-06-05

**Authors:** Inmaculada Galindo, Bruno Hernaez, Gema Díaz-Gil, Jose M. Escribano, Covadonga Alonso

**Affiliations:** Departamento de Biotecnología, Instituto Nacional de Investigación y Tecnología Agraria y Alimentaria (INIA), Autopista A6 Km 7, 28040 Madrid, Spain

**Keywords:** vBcl-2, viral Bcl-2, cBcl-2, cellular Bcl-2, ORF, open reading frame, HA, hemagglutinin tag, Bid, BH3 interacting domain death agonist, tBid, truncated Bid, ASFV, African swine fever virus., Apoptosis, Bid, Virus–cell interaction, African swine fever virus

## Abstract

Several large DNA viruses encode Bcl-2 protein homologues involved in the regulation of the cellular apoptosis cascade. This regulation often involves the interaction of these viral proteins with diverse cellular Bcl-2 family members. We have identified the specific interactions of A179L, an African swine fever virus (ASFV) Bcl-2 homologue, with the active forms of the porcine BH3-only Bid protein (truncated Bid p13 and p15). Transient expression of ASFV A179L gene in Vero cells prevented apoptosis induced by these active forms of Bid protein. Interestingly, A179L protein was able to interact, also with the main core Bcl-2 proapoptotic proteins Bax and Bak, and with several BH3-only proteins with selective binding restrictions for full length Bid and Noxa. These results suggest a fine regulation for A179L action in the suppression of apoptosis in infected cells which is essential for efficient virus replication.

## Introduction

Some of the best studied cellular apoptosis regulators belong to the Bcl-2 family, which include both proapoptotic and antiapoptotic effectors (Korsmeyer, 1995; White, 1996). Members of Bcl-2 family have common conserved regions, designated Bcl-2 homology regions 1, 2, 3 and 4 (BH1, BH2, BH3 and BH4). Bcl-2 protein is the prototypical member that negatively regulates apoptosis and contains all the Bcl-2 domains. This protein preserves mitochondrial integrity by interacting with Bcl-2 family proapoptotic members (Petros et al., 2004). Apoptosis inducer members include BH3-only proteins, which are cellular damage sensors that initiate rapidly the death process, and Bax-like proteins that act downstream of BH3-only proteins to permeabilise the mitochondrial outer membrane (Bouillet and Strasser, 2002).

The apoptosis cascade may be initiated by pathogenic agents such as viruses and is considered as part of the cellular defensive mechanism. Viruses have adapted numerous ways of circumventing this host defensive response, including regulation of endogenous host death receptors and ligands, expression of caspase activation inhibitors, regulation of host Bcl-2 proteins, and expression of viral homologues of Bcl-2 (vBcl-2s) (Benedict et al., 2002; Polster et al., 2004).These viral genes encoding proteins with amino acid sequence similarity to cellular Bcl-2 apoptosis inhibitors have been identified in several viral models including Epstein–Barr virus (EBV) (Bellows et al., 2002; Henderson et al., 1993), human herpes virus 8 (HHV8) (Cheng et al., 1997) and African swine fever virus (ASFV) (Afonso et al., 1996) between others. The role of these vBcl-2s in diverse aspects of the viral cell-cycle and their mechanism of action has been gradually emerging (Everett and McFadden, 1999; Hardwick and Bellows, 2003). VBcl-2s mediate inhibition of apoptosis in infected cells and prevent premature death of the host cell which would impair virus replication and might have also a role in the development of persistent infection (Cuconati and White, 2002).

African swine fever virus (ASFV) is a double stranded large DNA virus that induces an acute disease of swine in which apoptosis plays a central role in pathogenesis. Virus infection induces apoptosis in target and immune defence cells (Oura et al., 1998; Ramiro-Ibanez et al., 1996). This programmed cell death induction in the target cell has been recently tracked in vivo in living cells as infection progresses and the execution phase of apoptosis becomes evident at late infection times (Hernaez et al., 2006). In fact, ASFV encodes for various apoptosis inhibitor genes, one of these sharing high sequence similarity to cellular Bcl-2, the ASFV A179L gene (Revilla et al., 1997; Yanez et al., 1995). This gene encodes for a 21 kDa protein which is expressed at early and late times after infection and is essential for virus replication throughout the infection cycle (Brun et al., 1996; Neilan et al., 1993). A179L is highly conserved in most ASFV isolates, both in pathogenic and cell-cultured adapted isolates. In comparison to other viral Bcl-2s, its sequence is very similar to the cellular protein containing all the characteristic Bcl-2 homology domains (BH1, BH2, BH3 and BH4), but lacking the transmembrane region (Afonso et al., 1996). A179L is involved in the suppression of apoptosis in ASFV infected cells (Brun et al., 1996) and prolongs host cell survival until the replication of the large viral genome is completed. Prolonged cell survival could be relevant facilitating a persistent infection (Afonso et al., 1996; Brun et al., 1996). Moreover, given the fact that immune defence entails cytotoxic T lymphocytes attack against infected cells by TNFα, FasL or TRAIL, A179L could play a role in the infected cell escape to premature death due to cytokine signalling. Mutations in the BH1 domain of A179L abrogate its death-repressor activity (Revilla et al., 1997). Interestingly, this protein is functional and prevents virus induced apoptosis not only in mammalian cells but also in insect cells (Brun et al., 1998), indicating a very low degree of species-specificity, as would be required of a viral protein that should exert its function in both, mammals and the arthropod vector (White, 1996). The ASFV arthropod vectors are ticks of the *Ornithodorus* genus (Plowright et al., 1969).

However, the precise mechanism of action of A179L remains undefined. Some evidences suggest that most vBcl-2s might target the core cellular proapoptotic machinery for inhibition (Bellows et al., 2002; Nava et al., 1997), but also redundant actions on specific, short BH3 proapoptotic members have been described (Boyd et al., 1995; Han et al., 1996b) probably directed to secure apoptosis inhibition in the infected cell.

The aim of this work was to characterize the biochemical mechanisms by which the A179L protein suppresses apoptosis. Active forms of Bid protein from *Sus scrofa* were first identified as A179L interacting proteins. A179L blocked Bid-induced apoptosis when transfected in Vero cells, pointing out that A179L action may take place downstream of caspase 8 or granzyme B cleavage. In this work, we have shown that A179L protein interacted specifically with both BH3-only proapoptotic proteins and the core cellular proapoptotic machinery suggesting a central role for this protein in the inhibition of apoptosis induced by a wide variety of stimuli.

## Results

### Interaction of ASFV A179L protein with p13 truncated Bid protein

Although previous results demonstrated that ASVF A179L protects cells from programmed cell death, the molecular mechanisms supporting this biological effect remain to be determined. To elucidate the role of the virus Bcl-2 homologue, the yeast two hybrid system was used to screen a porcine macrophage cDNA library with full-length A179L as bait, searching for cellular interacting proteins. Two yeast clones were identified to induce the expression of the three reporter genes (*HIS3*, *LEU2*, *TRIP1*), as indicated by growth on SD medium and blue staining in the presence of X-α-Gal. These two cDNA clones were characterized by nucleotide sequence analysis. One of these clones was not included in these studies because the sequence revealed no significant homology to any known gene or translated product at the NCBI data base. Another cDNA clone was found to encode a porcine protein with high percentage homology (65%) to tBid-p13 protein from *Homo sapiens*. TBid-p13 protein corresponds to the carboxy-terminal fragment of Bid protein, named truncated Bid (tBid). This protein is related with apoptosis mediated by death receptors and results from post-transductional cleavage of full length Bid by caspase 8 or granzyme B (Li et al., 1998; Luo et al., 1998). The resulting protein of 13 kDa has been found to be one of the active forms of Bid protein inducing apoptosis (Gross et al., 1999a,b; Stoka et al., 2001).

**Fig. 1:**
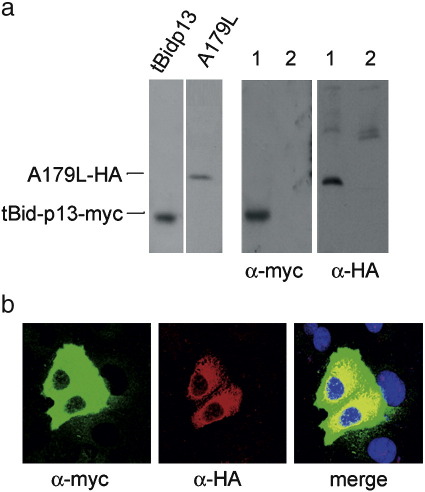
ASFV A179L interaction with porcine tBid-p13 in mammalian cells by affinity chromatography and confocal microscopy. (a) Total cell lysates from single transfected cells with either tBid-p13–myc or A179L–HA were blotted for anti-myc or anti-HA (left panel). Interaction of A179L with tBid-p13 was confirmed by immunoprecipitation of HA-tagged A179L and myc-tagged tBid-p13, from Vero cells transfected with the corresponding expression constructs. The immunoprecipitates were analyzed by SDS-PAGE and subjected to immunoblotting with antibodies against myc or HA. Lane 1, immunoprecipitation with a mAb against HA. Lane 2, immunoprecipitation with normal mouse serum (right panel). (b) Vero cells were transiently transfected with pCMVA179L–HA and pCMVtBid-p13–myc plasmids. Colocalization of A179L and tBid-p13 was assayed by confocal microscopy. A179L was detected with anti-HA-Alexa 594 (red) and tBid-p13 with anti-myc-FITC (green). Nuclei were stained with Hoechst 33258 (blue). Colocalization areas are depicted in yellow.

To further confirm the interaction of tBid-p13 and ASVF A179L we tested the association of both proteins by immunoprecipitation. Protein extracts from Vero cells expressing A179L–HA or tBid-p13–myc protein were incubated with protein A-sepharose, previously conjugated with anti-HA antibody or with an irrelevant rabbit serum. After extensive washing, bound proteins were analyzed by immunoblotting with anti-myc or anti-HA antibodies (Fig. 1a). Results showed that tBid-p13–myc was coimmunoprecipitated with A179L–HA by the anti-HA antibody, confirming the interaction between these two proteins (Fig. 1a, lane 1). As expected, tBid-p13–myc was not immunoprecipitated by the irrelevant rabbit serum (Fig. 1a, lane 2).

To gain further insight into the interaction between A179L and p13 truncated form of Bid protein, confocal microscopy assays were also performed in Vero cells transiently expressing both proteins. In agreement with the results found with the two-hybrid assay, we found colocalization of both proteins, A179L and p13 truncated form of Bid (Fig. 1b). The subcellular localization of porcine tBid, diffusely distributed throughout the cytoplasm, was similar to that of other reported Bid proteins (Wang et al., 1996). A179L stained diffusely the cytoplasm with a significant proportion accumulating in the perinuclear space. Colocalization of both proteins, A179L and tBid-p13, was found mainly in the perinuclear region as it is shown in the overlay (colocalization percentage over 72%). The above results indicate an interaction between ASFV A179L and porcine truncated Bid protein not only in vitro but also in vivo.

### Isolation and sequence analysis of cDNA encoding porcine Bid

On the basis of the information from NCBI data base, where the A179L interacting sequence showed a high degree of homology with one of the truncated forms of human Bid protein, a RACE-PCR was performed to obtain the complete sequence for porcine Bid protein. The full length sequence generated of porcine Bid gene was deposited at GenBank under accession no DQ087226.

**Fig. 2:**
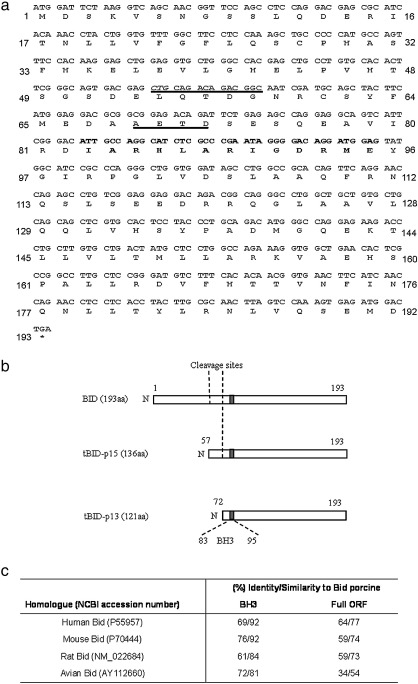
Complete sequence of porcine Bid protein. (a) Full length Bid open reading frame (ORF) cDNA clone and its predicted amino acid sequence. A conserved BH3 domain (bold) and putative cleavage sites (underlined) are indicated. (b) Schematic structure of porcine Bid isoforms, pointing out BH3 and protease cleavage domains. (c) Homology to other annotated Bid proteins. The identity/similarity values (%) were obtained from ClustalW program (http://www.ebi.ac.uk/clustalw/). Percentages of similarity for BH3 domain were calculated including in each BH3 domain the following amino acid residues: Human Bid aa 86–98, mouse Bid 85–97, rat Bid 87–99, avian Bid 87–99 and porcine Bid 83–95. For full open reading frame (ORF) comparation, the full-length protein for each species analyzed was included from the start codon to the stop codon.

This sequence predicts an open reading frame of 579 nucleotides encoding for a protein of 192 amino acids. The protein contains a BH3 domain from amino acid 83 to amino acid 95. Moreover, similarly to the human Bid sequence, the analysis of the porcine sequence indicated the presence of two potential cleavage sites present in the full length protein (Fig. 2a). The primary site (LQTDG), at 54–57 amino acids, is the putative caspase 8 and 1 cleavage sites which generate a 15 kDa truncated form of Bid protein (tBid-p15 protein). The secondary site (AETD) at residues 69–72, would be recognized by both caspase 8 and granzyme B, generating tBid-p13 protein. A third cleavage site generating tBid-p11 protein, described in *H. sapiens* as an inactive form of Bid (Gross et al., 1999a,b), was not found in the porcine Bid sequence however (Fig. 2b).

A comparison between the deduced amino acid sequences of porcine Bid with those from other organisms was performed using the computer analysis tool ClustalW. The porcine Bid sequence exhibited high identity with every annotated sequence from mammalian origin Bid proteins (Fig. 2c): *H. sapiens* (64%), *Mus musculus* (59%) and *Rattus novergicus* (59%). In contrast, porcine Bid was less closely related to avian Bid protein (*Gallus gallus*), with an identity of 34%. Alignment of Bid amino acid sequences indicates that the BH3 domain is highly conserved between species, sharing 75–60% identity (Fig. 2c). Cleavage sites were also conserved, particularly, primary cleavage site (LQTD) which was identical in all five species examined (100%). Secondary cleavage site (AETD) yielded lower identity percentage (50%) between the porcine Bid site and the homologue mammalian sequences.

### Apoptosis induction by porcine Bid truncated isoforms in Vero cells

On the basis of its homology with human and murine Bid proteins, we predicted that porcine Bid protein would also function as a proapoptotic protein. To investigate the proapoptotic activity of porcine Bid protein, as well as its truncated forms activities, we transfected Vero cells with either pCMVBid–myc, pCMVtBid-p13–myc, pCMVtBid-p15–myc or empty pCMV–HA (negative control) constructs and examined by immunofluorescence microscopy.

**Fig. 3:**
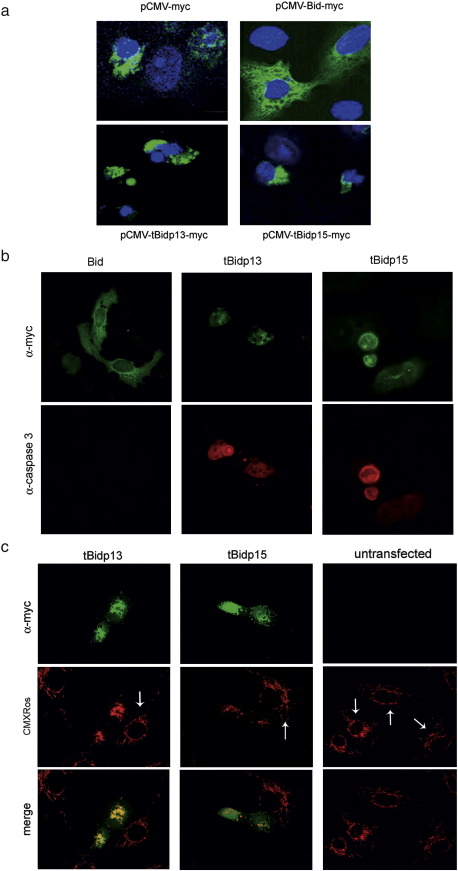
Proapoptotic activity of truncated Bid isoforms in Vero cells. (a) Vero cells were tranfected with either pCMV–myc (negative control), pCMVBid–myc, pCMVtBid-p13–myc or pCMVtBid-p15–myc. Full length and truncated forms of Bid, were detected using anti-myc-FITC (green). DNA was stained with Hoechst 33258 (blue). Cells transfected with active Bid forms showed nuclear features characteristic of apoptosis: nuclear size reduction, condensation and irregular chromatin pattern. In contrast, full length Bid transfection did not result in altered nuclear morphology. (b) Expression of truncated forms of Bid in transfected cells induced activation of caspase 3. (c) Vero cells expressing Bid truncated forms presented irregular mitochondrial clumping using a mitochondrial membrane potential-sensitive dye (CMXRos, orange). The finely reticular mitochondrial pattern is evident in non-transfected cells (arrows).

As described in other species, Vero cells expressing full length porcine Bid did not show evident apoptosis features or morphological changes up to 24 h after transfection. In contrast, characteristic nuclear features of apoptosis were found in those cells expressing tBid-p13 and tBid-p15. Condensation and fragmentation of chromatin, leading to typically small or shrunken nuclei were found in cells expressing truncated forms of Bid protein, whereas cells transfected with control vector and full length Bid exhibited intact round-shaped nuclei with diffuse bright blue fluorescence with Hoechst 33258 staining (Fig. 3a). In the same way, cells transiently expressing truncated forms of Bid, but not complete Bid, showed activation of caspase 3 (Fig. 3b). In order to further characterize apoptosis induced by the active forms of Bid protein, a potential-sensitive dye (CMXRos) was used to determine mitochondrial changes. Mitochondria stained finely distributed along the cytoplasm in healthy non-transfected cells. Nevertheless, cells transfected with the active forms of Bid proteins changed dramatically this distribution, presenting accumulation and irregular clumping (Fig. 3c). In these transfected cells, either tBid-p13 or tBid-p15, was found localizing with mitochondria. These morphological changes were easily observed in 96 and 92% of the cells transiently expressing tBid-p13–myc and tBid-p15–myc, respectively, from a total of 50 transfected cells examined.

Jointly, these results showed the proapoptotic activity of truncated porcine Bid proteins rapidly inducing apoptosis after transient transfection. In addition, the absence of proapoptotic activity of full length porcine Bid protein is in agreement with the notion that only caspase 8 or granzyme B post-transductional processing render this protein active.

### Inhibition of proapoptotic activity of truncated forms of Bid by specific interaction with A179L

Although sequence analysis and functional experiments showed that there are at least three isoforms of porcine Bid protein, only tBid-p13 protein, was at first identified as an ASFV A179L interacting protein when a yeast two hybrid screening was conducted using a porcine macrophage library. To determine if A179L was also able to interact with others forms of porcine Bid protein (full length Bid and tBid-p15), we performed a series of direct yeast two hybrid assays. Y190 yeast strain was cotransformed with pGBT9–A179L vector and pATC2 vector containing Bid, tBid-p13 or tBid-p15. The results (Table 1) indicated that A179L selectively interacts with active forms of Bid protein, tBid-p13 and tBid-p15, and failed to associate with full length Bid, the inactive form of Bid protein.

**Fig. 4:**
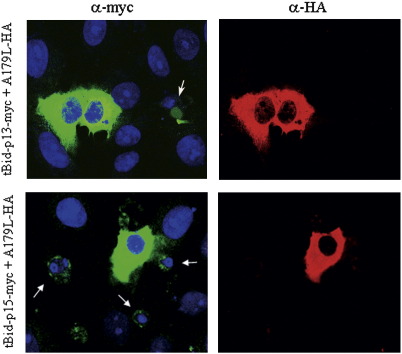
Proapoptotic activity in Vero cells expressing simultaneously ASVF A179L and truncated Bid proteins. Plasmids expressing pCMVtBid-p13–myc or pCMVtBid-p15–myc were cotransfected alternatively in Vero cells together with pCMVA179L–HA. An antibody against the myc epitope tag FITC conjugated (green) and anti-HA antibody conjugated with Alexa 594 (red) were used to detect truncated Bid and *A179L* proteins, respectively and Hoechst 33258 DNA dye stained nuclei in blue. Single transfected cells with truncated Bid underwent typical apoptosis changes with a marked size reduction (arrows) while cotransfected cells conserved normal morphology.

Once the interaction between A179L protein and the active forms of porcine Bid protein was found, we investigated the functional role of these interactions and whether A179L binding to truncated forms of Bid could interfere with the death signal mediated by these proteins. We first tested proapoptotic activity mediated by active Bid isoforms alone. For this purpose, Vero cells were cotransfected with pCMV–A179L–HA and pCMVtBid-p13–myc or pCMVtBid-p15–myc and were then analyzed by laser confocal microscopy. As it was shown above (Fig. 3), 24 h post-transfection, expression of tBid proteins induced apoptosis features. In contrast, every cell transiently expressing A179L and either tBid-p13 or tBid-p15 did not show apoptosis features; that is, double positive cells exhibited healthy cellular morphology (Fig. 4). DNA pattern evaluation in those single transfected cells expressing truncated Bid proteins revealed nuclei condensation and chromatin fragmentation (Fig. 4, indicated by arrows). Moreover, these cells exhibited rounding and nuclear size reduction. Jointly, these results suggest that A179L is able to impact on apoptotic pathway mediated by the BH3-only protein Bid, which is a central actor in the death receptor apoptosis pathway, protecting the host cell against programmed cell death.

### Interaction of A179L with Bcl-2 proapoptotic proteins

Many of the viral Bcl-2 homologues have been shown to inhibit apoptosis induced by a variety of cell death stimuli (Cuconati and White, 2002). Nevertheless, different death stimuli seem to activate different BH3-only effectors. To further investigate which pathways were inhibited by A179L, we tested if this protein was able to interact with other proapoptotic Bcl-2 family proteins (Table 1). Several mammalian BH3-only proteins, including murine Bid were cloned into pATC2 vector to be used in a yeast two-hybrid assay. Three isoforms of Bim (Bim S, Bim L and Bim EL) were analyzed, since differences in the proapoptotic potential activity of these isoforms have been previously described (Marani et al., 2002). *H. sapiens* Bik and its murine homologue (Biklk) were tested to analyze possible species specific differences.

All clones cotransformed with pGBT9-A179L and pATC2-BH3-only proteins, except those containing pATC2-Bid or pATC2-Noxa, were able to grow to form blue-stained colonies on synthetic media lacking three amino acids (Trp, Leu, His) and adenine. These results suggest that, as other apoptosis suppressors which are members of Bcl-2 family, A179L mediates inhibition through heterodimerization with BH3-only proteins. However, A179L failed to associate with murine complete Bid and human Noxa proteins. This result confirms previous data obtained for porcine Bid, indicating that A179L interacts only with active forms of Bid protein. Interestingly, this viral gene failed to associate with Noxa suggesting that A179L is not involved in the apoptosis pathway mediated by this BH3-only protein.

BH3-only proteins are not the only targets described for viral Bcl-2 homologues. Interactions with the core cellular proapoptotic machinery, represented by Bax and Bak have been identified for adenoviruses and herpesviruses among others (Cuconati and White, 2002). Thus, to determine if the mechanisms responsible for A179L mediated inhibition of apoptosis were related with the core cellular proapoptotic machinery, yeast two hybrid assays were performed using Bax and Bak as preys. As indicated in Table 1, A179L was found to interact with these two proteins.

To characterize the specificity of the A179L interactions, the ability of other ASFV proteins to interact with these Bcl-2 propapoptotic members was also determined. ASFV p30 (Afonso et al., 1992), p54 (Rodriguez et al., 1994) and MyD (Rivera et al., 2007) were chosen since these are major viral proteins upon infection. None of these three virus proteins were able to interact with Bcl-2 propapoptotic members in the yeast two hybrid assays.

These results indicated that A179L selectively interacts with functionally related Bcl-2 family members to inhibit apoptosis. Hence, mechanisms underlying this *A179L* inhibition seem to be related to heterodimerization with both the core apoptotic machinery and its upstream regulators, the BH3-only proteins.

**Fig. 5:**
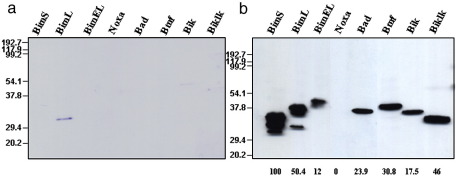
A179L interaction with BimS, BimL, BimEL, Bad, Bmf, Bik and Biklk but not with Noxa, by GST-pull down assays. Bacterial cell lysates containing His-tagged BimS, BimL, BimEL, Bad, Bmf, Bik, Biklk or Noxa were incubated with equal amounts of GST (a) or GST–A179L (b) attached to glutathione matrix beads. After extensive washing, proteins bound to beads were resolved by SDS-PAGE and inmunoblotted for anti His. The positions in Kilodaltons of protein standards are shown to the left of the gel. Bands were quantified by densitometry using TINA software package (Raytest) and the percentage of protein in each lane, relative to BimS, is shown below.

To confirm the interactions between A179L and the Bcl-2 proapoptotic proteins, previously identified by yeast two hybrid assays, we tested most of these interactions *in vitro* using recombinant proteins. A recombinant vaccinia virus expressing A179L protein fused to glutathione *S*-transferase (GST–A179L) was constructed. Several Bcl-2 proapoptotic proteins were expressed in *Escherichia coli* as fusion proteins with polyhistidine tag (His–BH3-only proteins). Expression levels of His–BH3-only proteins in *E.coli* were assessed by Western blot (data not shown). Equal amounts, as judged by Coomassie blue staining, of recombinant GST–A179L and GST were immobilized on glutathione-sepharose beads and incubated with bacterial cell extracts containing the His–BH3-only proteins. After extensive washing, bound proteins to the G-Sepharose beads were separated by SDS-PAGE and visualized by Western blotting with a monoclonal antibody recognizing the His-tag. A179L interacted with BimS, BimL, BimEL, Bad, Bmf, Bik and Biklk. An interaction between A179L and Noxa was not detected, confirming previous results obtained in yeast two hybrid assay. An estimation of the percentages of BH3-only proteins interacting with A179L and relative to BimS is shown in Fig. 5. The control experiments showed that GST alone did not interact with the Bcl-2 proapoptotic proteins analyzed.

## Discussion

Apoptosis represents an important innate cellular mechanism for curtailing virus infection, and many viruses have in turn, developed strategies for inhibiting or delaying this cellular response (Benedict et al., 2002). This represents a mechanism used by the host immune system and the infected host cell itself as part of the antiviral response but often contributes to pathogenesis (Barber, 2001; Hardwick, 2001). For a successful replication, viruses must modulate apoptotic pathways to extend the lifespan of their host cell and encode homologues of antiapoptotic Bcl-2 proteins to this end (Hardwick and Bellows, 2003). A179L is one of those viral Bcl-2 homologues that protect cell from programmed cell death (Brun et al., 1996). Various viral Bcl-2 homologues have been demonstrated to interact with the core cellular proapoptotic machinery for inhibition, but it was also proposed that these virus genes may target BH3-only proteins, perhaps to secure a broad spectrum of apoptosis inhibition to the infected cell. Recently, vaccinia virus N1 protein, a novel Bcl-2-like antiapoptotic protein, has been shown to interact with Bid, Bad and Bax (Cooray et al., 2007). Alternatively, it may be so critically important to block apoptosis by death receptor ligands that viruses encode redundant inhibitory mechanisms to ensure survival of the infected cell. The first indication that the adenovirus Bcl-2 E1B 19K protein functions similarly to cellular Bcl-2 emerged when a yeast two-hybrid screening using E1B 19K as bait, identified Bax and Bak as interacting proteins (Farrow et al., 1995; Han et al., 1996a,b). Nevertheless, searches in databases do not reveal homology between adenovirus E1B 19K and Bcl-2. On this basis, E1B 19K is referred to as a “functional homologue” of Bcl-2 and the three dimensional structure revealed a folded structure similar to Bcl-2 (Polster et al., 2004). Some other viral Bcl-2 homologues share low amino acid sequence similarity with cellular Bcl-2 such as murine γ-herpes virus 68 (γHV68) M11 protein (Wang et al., 1999). Viral Bcl-2 homologues sharing the four homology domains BH1–BH4 include human herpes virus 8 Bcl-2 (Cheng et al., 1997), fowl poxvirus (FPV039) (Banadyga et al., 2007) and ASFV A179L (Neilan et al., 1993). The last one, lacking a putative membrane-spanning region (Afonso et al., 1996).

Although the role of most of these viral Bcl-2 homologues in infection have been gradually emerging (Cuconati and White, 2002), the biology of Bcl-2 homologue encoded by African swine fever virus is poorly understood. This is related in part to the complex genetic manipulation of this virus and the lack of convenient cell culture system and/or animal models (Hardwick and Bellows, 2003). In this work, using a porcine macrophage cDNA library, by yeast two-hybrid screening with the A179L protein as bait, we identified a truncated form of porcine Bid as the first cellular interacting protein characterized. It is known that after death receptors activation, the BH3-only Bid protein is proteolysed by caspase 8 (Li et al., 1998), whereas Bid is proteolysed by granzyme B during granule-mediated cytotoxic T lymphocyte cell killing (Heibein et al., 2000). These post-transductional modifications are necessary for function and then truncated products are translocated to the mitochondria where they promote the exit of cytochrome *c* (Fleischer et al., 2003). We have described *S. scrofa* Bid and its truncated forms encoding sequences, and according to the reported data for human Bid only these truncated forms resulted proapoptotic. Putative cleavage sites for caspase 8 and granzyme B were found in the porcine Bid sequence that would generate two carboxy-terminal fragments of 15 kDa (tBid-p15) and 13 kDa (tBid-p13), similarly to human Bid. According to previous results (Fleischer et al., 2003; Zha et al., 2000) we have shown that only truncated Bid forms caused an efficient Bid-mediated cell death, demostrating that a viral Bcl-2 is able to prevent this process. Moreover, Bid is constitutively phosporylated and must be dephosphorylated for inducing apoptosis. Several residues of Bid are phosphorylation targets for casein kinases I and II, being then insensitive to caspase 8 cleavage (Desagher et al., 2001). Similarly to previously described Bid proteins, serine residues present at porcine Bid protein also suggests a similar regulation by phosphorylation status.

An intriguing fact was to find that full length Bid failed to interact with A179L*,* given that the BH3 region is present in the linear sequence of this protein. The solution structure of the Bid protein was determined using NMR spectroscopy (Chou et al., 1999; McDonnell et al., 1999). The structure of full-length Bid in solution consists of eight α-helices arranged with two central somewhat more hydrophobic helices forming the core of the molecule. The third helix, which contains the BH3 domain, is connected to the first two helices by a long flexible loop, which includes the caspase-8 cleavage site. After caspase cleavage, the activated fragment, tBid, lacks the first helix, the small additional helix, and part of the unstructured loop. Since the first α-helix of Bid has hydrophobic interactions with the BH3 region in the native protein, removal of this helix would expose a large hydrophobic surface on the BH3 helix, making it accessible for binding by other Bcl-2 family members (Gong et al., 2004; Petros et al., 2004).

Bid is the first molecule likely to be involved in A179L pathway since we showed that, in transfected cells, A179L is able to prevent Bid-induced apoptosis. This result indicates that ASFV possibly inhibits the death receptor apoptosis pathway downstream of caspase 8 or granzyme B activation which would cleave Bid in its active forms and suggests then that one possible function of A179L could be to prevent death of the infected cell due to signaling by death cytokines. There are several examples of inhibition of death receptor signaling by vBcl-2 proteins. The BHRF1 and Balf1 proteins of EBV are capable of inhibiting TNF-α and FasL induced cell death (Foghsgaard and Jaattela, 1997; Marshall et al., 1999). The M11 protein of γHV68 can inhibit TNF-α and FasL induced cell death (Wang et al., 1999) and the vBcl-2 of herpes virus saimiri (HVS) blocks Fas signalling in a cell-type dependent manner (Derfuss et al., 1998; Feng et al., 2004). Those examples indicate how crucial is for infected cells to escape the attack of cytokine signaling triggered by cytotoxic T cells. The presence of redundant antiapoptotic functions in many viral genomes makes unclear what is the overall contribution of vBcl-2 proteins in the inhibition of cytokine death signaling during infection.

Interestingly, A179L is also capable of interacting with Bax and Bak proapoptotic proteins and with most of the BH3-only proteins described. This suggests that A179L protein acts as a receptor for the BH3 domain of proapoptotic Bcl-2 family proteins and is able to antagonize their function. In doing this, the ASFV Bcl-2 homologue is predicted to function by similar biochemical mechanisms to cellular Bcl-2 (cBcl-2), namely by interacting with other family members, and by either promoting or antagonizing the function of its binding partner (Gross et al., 1999a). These protein–protein interactions rely on the BH3 domain of one protein, interacting with the hydrophobic cleft created by BH1–BH3 domains of cBcl-2 (Sattler et al., 1997). This may explain why BH1 domain mutations in ASFV Bcl-2 homologue eliminate its proapoptotic activity (Revilla et al., 1997).

Thus, cellular and viral Bcl-2 and perhaps other viral antiapoptotic proteins could act both by inhibiting Bak/Bax and by sequestering BH3-only death molecules. However, the physiological context of these activities still remains to be determined. It has been postulated that a possible preference of viral Bcl-2 homologues for Bak and Bax versus BH3-only proteins may vary their regulation and expression in different cell types (Cuconati and White, 2002). As Polster et al. suggest (Polster et al., 2004), it is difficult to conclude that vBcl-2 proteins primarily target the core apoptotic machinery rather than their upstream BH3 activators, since the majority of the studies to date have been focused on Bax and Bak and rarely covered an extensive study of vBcl-2 interactions with the different BH3-death agonist molecules. Data presented in this work for ASFV, suggest a relevant role for BH3-only molecules as A179L targets. An interesting finding is that full length Bid and Noxa have been the only exceptions which did not interact with A179L. This differential targeting of BH3-only proteins has been reported for the cellular prosurvival Bcl-2 family members similarly, and Noxa also presented a more restrictive binding (Chen et al., 2005). Noxa is highly specific for the antiapoptotic Bcl-2 family members Mcl-1 and Bfl-1/A1 being relevant for a fine tuning of survival/cell death pathways with potential therapeutic applications.

In conclusion, we have shown the direct interaction of ASVF A179L with the active forms of porcine Bid protein (tBid-p15 and p13) and that A179L was able to suppress mitochondrial apoptotic signaling pathway initiated by these active Bid proteins. We have also found A179L interactions with several Bcl-2 family proapoptotic members, suggesting a pivotal role for this virus Bcl-2 homologue in the regulation of apoptosis during virus infection. These results indicate that ASFV A179L protein could block apoptosis through interaction with specific Bcl-2 proapoptotic proteins. As a consequence, the abrogation of death receptor signaling through Bid could allow the infected cell to escape immune surveillance by rendering the cell resistant to the action of TNF-α, for instance. But as with most DNA viruses, the activity of A179L during infection is only part of a multilayered response to apoptosis induction from the different antiapoptotic ASFV genes that apparently act to block a wide spectrum of molecules and at different levels of distinct apoptotic pathways (Hernaez et al., 2004b). Future work will be directed to the elucidation of the role of the many ASFV interactions with cell death related proteins and their relevance along infection.

## Materials and methods

### Cell culture, viruses and transfection

Vero, BSC-1 and CV-1 cell lines were obtained from the American Type Culture Collection (ATCC). Cells were cultured in Dulbecco's modified Eagle's medium supplemented with 5% calf fetal serum, 100 IU/ml penicillin, and 100 μg/ml streptomycin.

BA71V is the prototype ASFV strain used in our laboratory and represents a high cell-passage number strain. Preparation of viral stocks, titrations, and infection experiments were carried out in Vero cells as previously described (Enjuanes et al., 1976; Hernaez et al., 2004a,b). Viral DNAs preps were extracted from infected Vero cells. Vaccinia virus vRB12 was made available by R. Blasco. Vaccinia virus infections were performed with 2% FBS in BSC-1 cells.

Vero cells grown to 40–50% confluence in 6-well culture dishes were transfected using FUGENE6 (Roche) and 2 μg DNA/10^6^ (ratio 1:6), following manufacturer's recommendations.

### RNA isolation, RT-PCR and DNA RACE-PCR

Total RNA was isolated using Trizol reagent (Invitrogen). Reverse Transcription Polymerase Chain Reaction (RT-PCR) amplification was carried out using Reverse Transcription System (Promega) according to the protocol recommended by manufacture. To obtain the sequence information for porcine Bid, we applied the method of Rapid Amplification of cDNA Ends (RACE) PCR (SMART™ RACE cDNA Amplification Kit, Clontech) following the manufacture's directions. RACE-PCR was carried on using SMART primer (Clontech) and a specific primer based on the 5′ end of porcine Bid protein: 5′-TCAGTCCATCTCACTTTGGACTAAG-3′.

### Vectors

#### Mammalian expression vectors

Plasmids expressing Bid, tBid-p13 or tBid-p15 were generated by RACE-PCR amplification of porcine macrophage total RNA to incorporate restriction sites (Supplementary table), followed by ligation of the amplified cDNA fragments with pCMV–myc plasmid (Clontech). The *A179L* gene was amplified by PCR from purified BA71V DNA, using oligonucleotides containing restrictions sites (supplementary table), and subcloned into pCMV–HA vector (Clontech).

#### Yeast expression vectors

Full length cDNAs encoding A179L, p54, p30 and MyD ASFV proteins were isolated from purified BA71V DNA and cloned into the pGBT9 vector (Clontech). Bax (GenBank accession no AY217036) and Bak (GenBank accession no NM_001188) cDNA were obtained by RT-PCR from human PBLs total RNA. Full length porcine Bid protein and its truncated forms were cloned into the pATC2 vector (Clontech). DNAs corresponding to BH3-only proteins were obtained from pEFFEE plasmids, kindly provided by Drs. Huang and P. Bouillet (Melbourne, Australia). The PCR products of Bcl-2 proapototic members were digested and cloned into the BamHI/EcoRI sites of pATC2 vector except for pATC2-Bax vector in which SmaI/EcoRI sited were used. Primers used for plasmid generation are listed in the supplementary table. All these constructs were sequenced to ensure that no errors were introduced.

#### Pull down assays vectors

Plasmid pRBgA179L used for the construction of a recombinant vaccinia virus expressing GST gene fused to the N terminus of the A179L protein was obtained by sequential cloning. The coding sequence of the A179L gene was amplified by PCR, using the genome of BA71V as template and oligonucleotides A179B (5′-AATATAGGGATCCGCTATGGAGGG-3′) (BamHI site underlined) and A179X (5′-GTAAAATCCTGCGCTCGAGCTATATC-3′) (XhoI site underlined). The PCR product was digested with BamHI and XhoI and inserted into BamHI/XhoI digested pGEX4T3 (Amersham Pharmacia Biotech) to generate pGEX-A179L.

The fusion gene GST–A179L was amplified by PCR from plasmid pGEX–A179L with oligonucleotides GSTNhe (5'-CACACAGGCTAGCGTATTCATGTCC-3') (NheI site underlined) and 179Hin (5'-GTCAGTCACGAAGCTTCCGCTCGA-3') (HindIII site underlined). The PCR product was cut with NheI and HindIII and inserted into NheI/HindIII digested pRB21 plasmid (Blasco and Moss, 1995) to generate pRB21g–A179L.

The coding sequences of the BH3 only proteins were amplified by PCR using the corresponding pATC2–BH3 only proteins vectors as templates and the oligonucleotides in supplementary table. The PCR products were digested and cloned into the NdeI/XhoI sites of Pet-19b vector (Novagen) fused to the C-terminus of the His-tag.

### Construction of a vaccinia recombinant virus

Vaccinia recombinant virus was isolated following infection/transfection experiments. CV-1 cells were infected with vRB12 at 0.05 PFU per cell and transfected 1 h later with pRBg-A179L plasmid by use of Fugene6 transfection reagent following the manufacturer's recommendations. Recombinant virus (VVgA179L) was isolated from progeny virus by rounds of plaque purification on BSC-1 cells by selecting large virus plaques following protocols described previously (Blasco and Moss, 1995).

### Immunofluorescence

Vero cells seeded in 24-well chamber slides were transfected with pCMV–myc and/or pCMV–HA constructions previously described. At 24 h post transfection, cells were fixed with acetone: methanol 1:1 for 2 min at − 20°C. Mouse monoclonal antibodies anti-myc-FITC (Babco) and anti-HA-Alexa 594 (Babco) were used at 1:25 dilution and 1:300 dilution, respectively. Mitochondrial staining was carried out using a potential-sensitive dye, chlorometil rosamine (CMXRos, Molecular Probes) and nuclei were stained with Hoechst 33258 (Sigma). Caspase 3 activation was determined using C8487 rabbit polyclonal antibody (Sigma) which exclusively recognizes the active form of caspase 3. After washing, coverslips were finally mounted on glass plates and cells were observed by confocal laser scanning microscopy in a Radiance 2100 MRC1024 (Bio-Rad) mounted on a Nikon Eclipse 300 microscope. Stadistical analysis of colocalization was performed using Lasersharp Processing 3.2 program (Bio-Rad).

### Immunoprecipitation

Twenty four hours post-transfection, Vero cells expressing pCMVtBid-p13–myc or pCMV–A179L–HA were lysed using non denaturing lysis buffer (50 mM Tris–HCl, pH 7.4, 300 mM NaCl, 5 mM EDTA, 0.1% Triton X-100) and a protease inhibitor cocktail (Roche), at 4°C. Cells were scraped, clarified by centrifugation and supernatants were mixed. Previously, Protein-A Sepharose (GE, Healthcare) was conjugated with anti-HA antibody (Babco) or irrelevant rabbit serum as negative control, for 16 h at 4°C. Supernatants were incubated with conjugated beads for 4 h at 4°C and gently washed with non denaturing lysis buffer. Bound proteins were eluted by boiling in SDS sample buffer and resolved on a 10% SDS-PAGE gel. Proteins were transferred onto a polyvinylidene difluoride membrane and probed with monoclonal anti-myc (Clontech) and anti-HA (Babco) antibodies. Proteins were detected using rabbit anti IgG-HRP (Bio-rad) as secondary antibody.

### Yeast two hybrid assays

For the yeast two-hybrid system, the plasmids pGBT9 and pATC2 (Clontech) were used as sources of the GAL-4 DNA-binding domain and GAL-4 transcriptional activation domain, respectively. All materials used for the analysis were derived from MATCHMAKER GAL-4 Two-Hybrid System (Clontech). Yeast cells were grown on YPD (1% yeast extract, 2% peptone, 2% dextrose, 2% agar for plates) or in synthetic minimal medium (0.143% yeast nitrogen base, the appropriate auxotrophic supplements) containing 2% of dextrose. Y190 strain carrying *His3* and *lacZ* as reporter genes was transformed with appropriate plasmids by the lithium acetate method (Gietz and Woods, 2006). The transformants were selected on the appropriate synthetic medium (SD/-Trp/-Leu/-His) and tested by colon-lift filter assay β-galactosidase activity with 5-bromo-4-chloro-3-indolyl β-d-galactopyranoside as substrate.

### Pull down assays

For preparation of GST, and His-BH3 only proteins *E. coli* strain BL21(DE3) cells, transformed with bacterial expression vectors pGEX-4T3 and Pet-19b–BH3-only proteins, respectively, were grown in LB medium supplemented with 50 µg/ml ampicillin to an OD600 nm of 0.4 to 0.6 at 37 °C. Subsequently, isopropyl-ß-d-thiogalactopyranoside (IPTG) was added to the cell culture at a final concentration of 1 mM, and incubation continued for an additional 4 h. Cells were harvested by centrifugation, suspended in 5 ml of lysis buffer (phosphate-buffered saline [PBS], 1% Triton X-100), and sonicated on ice. For preparation of GST–A179L, BSC1 cells were infected with VVgA179L virus at five PFU per cell. At 24 h postinfection, the cells were scraped and suspended in lysis buffer, sonicated on ice and spun down by centrifugation.

GST–A179L and GST proteins were purified by mixing with glutathione-Sepharose 4B beads (GE, Healthcare) for 2 h at 4 °C. Beads were washed with PBS. For the GST-based interaction assay, equal amounts of GST–A179L or GST, attached to glutathione matrix beads were incubated over-night at 4 °C in a tube rotator with bacterial cell lysate, containing expressed His-tagged BH3-only proteins, in binding buffer (50 mM HEPES, pH 7.5, 50 mM NaCl, 0.1% NP-40 with protease inhibitor mixture (Roche Molecular Biochemical). Beads were extensively washed four times with binding buffer. The glutathione-Sepharose beads immunoprecipitates were resuspended in denaturant buffer, boiled for 5 min at 95 °C, resolved by SDS-PAGE, blotted onto nitrocellulose membranes and probed with mouse monoclonal anti-6×His (catalog no. 8916-1; Clontech) before detection by enhanced chemiluminescence (ECL kit; Amersham).

## Figures and Tables

**Table 1:** Interaction of ASVF proteins A179L, p30, p54 and MyD with mammalian Bax, Bak and BH3 only proteins, as judged by yeast two hybrid assays

	A179L	p54	p30	MyD
Bid (Ss)	−	−	−	−
tBid-p15 (Ss)	+	−	−	−
tBid-p13 (Ss)	+	−	−	−
Bid (Mm)	−	−	−	−
Bad (Mm)	+	−	−	−
Bmf (Mm)	+	−	−	−
Noxa (Hs)	−	−	−	−
Puma (Hs)	+	−	−	−
DP5 (Mm)	+	−	−	−
Bik (Hs)	+	−	−	−
Biklk (Mm)	+	−	−	−
Bim S (Mm)	+	−	−	−
Bim L (Mm)	+	−	−	−
Bim EL (Mm)	+	−	−	−
Bax (Hs)	+	−	−	−
Bak (Hs)	+	−	−	−

Plasmids expressing viral proteins A179L, p30, p54 or MyD fused to GAL4 DNA-binding domain were cotransfected with plasmids expressing each Bcl-2 proapoptotic members fused to the GAL4 transcriptional activation domain. Protein-protein interaction (+) resulted in growth of yeast in absence of leucine, trytophan and histidine and blue staining in presence of X-Gal. Interaction was negative (−) with full length Bid, Noxa and irrelevant viral proteins included as controls. Ss: *Sus scrofa*, Mm: *Mus musculus*; Hs, *Homo sapiens*.
