# Supplementary material for: A179L, a viral Bcl-2 homologue, targets the core Bcl-2 apoptotic machinery and its upstream BH3 activators with selective binding restrictions for Bid and Noxa
Source: Virology. 2008 Jun 5;375(2):561–72. doi: 10.1016/j.virol.2008.01.050 (PMC2572728; doi:10.1016/j.virol.2008.01.050)
Supplement: Supplementary file 1 [file mmc1.doc]

| **Construct name** | **Forward primer (5’-3’)** | **Reverse primer (5’-3’)** | **Restriction sites** | **Template** |
| --- | --- | --- | --- | --- |
| **pCMVA179L-HA** | AGATCTGGGAGGGAGAAGAG | CTCGAGCTATATCAAATTGC | *Bgl*II/*Xho*I | Purified BA71 |
| **pCMVBid-myc** | GCGAATTCTGATGGATTCTAAGGT | GCGCGGCCGCTCAGTCCATCTCAC | *Eco*RI/*Not*I | Porcine macrophage RNA |
| **pCMVtBid-p13-myc** | AGATCTGGCAGATTCTGAG | GCGCCTCGAGTCAGTCCATCTCAC | *Bgl*II/*Xho*I | Porcine macrophage RNA |
| **pCMVtBid-p15myc** | GCGAATTCGGCAGATTCTGAG | GCGCCTCGAGTCAGTCCATCTCAC | *Eco*RI/*Xho*I | Porcine macrophage RNA |
| **pGBT9-A179L** | AAAGAATTCATGGAGGGAGAA GAG | AAAGGATCCCTATTCAAATTGC | EcoRI/BamHI | Purified BA71 |
| **pGBT9-p30** | AAAGAATTCATGGATTTTATTT  TAAATATATCCATG | AAAGGATCCATCCAATCATATA  AGAATAACTAAAAC | EcoRI/BamHI | Purified BA71 |
| **pGBT9-p54** | GCGCGAATTCTCTTCAAGAAA  GAAAAAAGC | GCGCGGATCCTTACAAGGAGTT  TTCTAGGTCTT | EcoRI/BamHI | Purified BA71 |
| **pGBT9-MyD** | GAATTCATGGGGGGGCGGC | GGATCCCTGCTGCTCCAGTAG | EcoRI/BamHI | Purified BA71 |
| **pATC2-Bim L** | CGGGATCCCGATGGCCAAGC  AAC | GGAATTCTCAATGCCTTCTCC | BamHI/EcoRI | pEFFE-Bim L (mm) |
| **pATC2-Bim EL** | CGGGATCCCGATGGCCAAGC  AAC | GGAATTCTCAATGCCTTCTCC | BamHI/EcoRI | pEFFE-Bim EL (mm) |
| **pATC2-Bim S** | CGGGATCCCGATGGCCAAGC  AAC | GGAATTCTCAATGCCTTCTCC | BamHI/EcoRI | pEFFE-BimS (mm) |
| **pATC2-Bad** | CGGGATCCCGTTCCAGATCC  CAGAG | GGAATTCTCACTGGGAGGGGG  TGG | BamHI/EcoRI | pEFFE-Bad (mm) |
| **pATC2-Bid** | CGGGATCCCGATGGACTCTG  AGGT | GGAATTCTAGTCCATCTCGTTT  CTA | BamHI/EcoRI | pEFFE-Bid (mm) |
| **pATC2-pBid** | GCGAATTCTGATGGATTCTAA  GGT | GCGCCTCGAGTCAGTCCATCTC  AC | EcoRI/XhoI | Porcine macrophage  RNA |
| **pATC2-tBid-p13** | GCGAATTCCTGGCAATCGATG  CAG | GCGCCTCGAGTCAGTCCATCTC  AC | EcoRI/XhoI | Porcine macrophage  RNA |
| **pATC2-tBid-p15** | GCGAATTCGGCAGATTCTGAG | GCGCCTCGAGTCAGTCCATCTC  AC | EcoRI/XhoI | Porcine macrophage  RNA |
| **pATC2-Bmf** | CGGGATCCCGATGGAGCCAC  CTC | GGAATTCTCACCAGGGCCCCA  CC | BamHI/EcoRI | pEFFE-Bmf (mm) |
| **pATC2-Bik** | CGGGATCCCGATGTCTGAAG  TAAG | GGAATTCTCACTGAGCAGCAGG  TG | BamHI/EcoRI | pEFFE-BiK (hs) |
| **pATC2-Biklk** | CGGGATCCCGATGTCGGAGG  CGA | GGAATTCTCACTGAAGCTGCAA  ATA | BamHI/EcoRI | pEFFE-Biklk (mm) |
| **pATC2-Noxa** | CGGGATCCCGATGCCCGGGA  GAA | GGAATTCTCAGGTTACTAAATT  GAA | BamHI/EcoRI | pEFFE-Noxa (hs) |
| **pATC2-Puma** | CGGGATCCCGATGCCCGTGT  C | GGAATTCCTACGCGCTCCGCCT  G | BamHI/EcoRI | pEFFE-Puma (hs) |
| **pATC2-DP5** | CGGGATCCCGATGGCCCGCG  CAC | GGAATTCTAATTGGGCTCCATC  TCG | BamHI/EcoRI | pEFFE-DP5 (mm) |
| **pATC2-Bax** | TCCCCCGGGGGAATGGACGG  GTCCGGGGAG | GGAATTCTCAGCCCATCTTCTT  CCA | SmaI/EcoRI | PBLs RNA |
| **pATC2-Bak** | CGGGATCCCGATGGCTTCGG  GGCAAGGC | GGAATTCTCATGATTTGAAGAA  TCT | BamHI/EcoRI | PBLs RNA |
| **Pet-BimEL** | CGCGCATATGGCCAAGCAACCTTCTG | CGCGCTCGAGTCAATGCCTTCTCCATAC | *NdeI/XhoI* | pATC-BimEL |
| **Pet-Bik** | CGCGCATATGTCTGAAGTAAGACCCCTC | CGCGCTCGAGTCACTTGAGCAGCAGGTG | *NdeI/XhoI* | pATC-Bik |
| **Pet-Biklk** | CGCGCATATGTCGGAGGCGAGACTTATG | CGCGCTCGAGTCACTGAAGCTGCAAATAC | *NdeI/XhoI* | pATC-Biklk |
| **Pet-BimL** | CGCGCATATGGCCAAGCAACCTTCTG | CGCGCTCGAGTCAATGCCTTCTCCATAC | *NdeI/XhoI* | pATC-BimL |
| **Pel-BimS** | CGCGCATATGGCCAAGCAACCTTCTGA | CGCGCTCGAGTCAATGCCTTCTCCAT | *NdeI/XhoI* | pATC-BimS |
| **Pet-Noxa** | CGCGCATATGCCCGGGAGAAAGGCGCG | CGCGCTCGAGTCAGGTTACTAAATTG | *NdeI/XhoI* | pACT-Noxa |
| **Pet-Bad** | CGCGCATATGTTCCAGATCCCAGAGTTT | CGCGCTCGAGTCACTGGGAGGGGGTG | *NdeI/XhoI* | pACT-Bad |
| **Pet-Bmf** | CGCGCATATGGAGCCACCTCAGTGTGT | CGCGCTCGAGTCAccagggccccAC | *NdeI/XhoI* | pACT-Bmf |

**Supplementary table**: Plasmid constructs generated in this work. Forward and reverse primers used for PCR amplification are indicated and cloning sites are underlined.
